# The Management of Poly-L-Lactic Acid (PLLA) Nodules: A Scoping Review and Evidence-Mapped Clinical Pathway

**DOI:** 10.7759/cureus.110742

**Published:** 2026-06-12

**Authors:** Julio César Flores Rodríguez, Brenda Mariel Porras Zamora, Nuria Montserrat Rico Macías, Lorena Valdovinos Martínez, Lilia Yolanda Camacho Frausto, Natalia Lorena Rossiere Echazarreta, César Octavio López Romero, Leobardo Velázquez Arenas, Jorge E. Krasovsky Santamarina, Rodrigo Merino Arellano

**Affiliations:** 1 Aesthetic and Regenerative Medicine, Clínica Aura, Monterrey, MEX; 2 Aesthetic and Regenerative Medicine, Sociedad Mexicana de Investigación en Medicina Estética (SOMIME), Monterrey, MEX; 3 Aesthetic and Regenerative Medicine, Consultorio Particular Dra. Brenda Mariel Porras Zamora, Mexico City, MEX; 4 Aesthetic and Regenerative Medicine, Sociedad Mexicana de Investigación en Medicina Estética (SOMIME), Mexico City, MEX; 5 Aesthetic and Regenerative Medicine, Clínica Dra. Nuria Rico, Tijuana, MEX; 6 Aesthetic and Regenerative Medicine, Sociedad Mexicana de Investigación en Medicina Estética (SOMIME), Tijuana, MEX; 7 Aesthetic and Regenerative Medicine, Consultorio Particular Dra. Lorena Valdovinos Martínez, Zapopan, MEX; 8 Aesthetic and Regenerative Medicine, Sociedad Mexicana de Investigación en Medicina Estética (SOMIME), Zapopan, MEX; 9 Dermatology, Healthskin, Mexico City, MEX; 10 Dermatology, Consultorio Particular Dra. Natalia Lorena Rossiere Echazarreta, Huixquilucan, MEX; 11 Plastic and Aesthetic/Reconstructive Surgery, Innovare Especialidades Quirúrgicas y Cirugía Plástica, Zapopan, MEX; 12 Dermatology, Filokalia, Monterrey, MEX; 13 Plastic and Aesthetic/Reconstructive Surgery, Berclinic, Mexico City, MEX; 14 Plastic Surgery, TecSalud, San Pedro Garza García, MEX

**Keywords:** adverse event, clinical pathway, filler complication, granuloma, injectable filler, nodule, poly-l-lactic acid (plla), sculptra

## Abstract

Poly-L-lactic acid (PLLA) is a collagen-stimulating, biodegradable filler used for facial rejuvenation and body contouring. Though relatively safe, PLLA-related nodule formation and granulomatous reactions remain significant clinical complications with no adequate evidence-based management guidelines. This scoping review aims to summarize the available literature on the prevention, diagnosis, classification, and management of PLLA-associated nodules and to propose an evidence-based management pathway. The present study conducted a PRISMA-ScR (Preferred Reporting Items for Systematic Reviews and Meta-Analyses extension for Scoping Reviews)-compliant scoping review by searching PubMed/MEDLINE, Scopus, Web of Science, and the Cochrane Library from 2000 through April 2026. Inclusion criteria included all studies reporting PLLA-related nodules. A total of 40 studies were identified as eligible, including case reports, case series, retrospective cohorts, RCTs, systematic reviews, and expert consensus statements. Nodule formation appeared to be linked to various factors such as the dilution protocol, injection technique, reconstitution method, and injection depth. Clinical phenotype identification revealed four phenotypes: non-inflammatory, inflammatory-immunologic, infectious-biofilm, and other unusual presentations. Useful diagnostic aids included ultrasound and high-frequency ultrasound, and histopathological confirmation was recommended for atypical presentations. Various management techniques included conservative management, intranodular corticosteroids, 5-fluorouracil, antibiotics, immunotherapy, and surgery. The present study proposes a four-phenotype classification and a six-step evidence-based management algorithm for PLLA nodule presentation. Effective management appears to begin with prevention.

## Introduction and background

Poly-L-lactic acid (PLLA) is a manufactured, biodegradable alpha-hydroxy acid polymer and is classified as a collagen biostimulator. Following its European clearance under the brand name NewFill and FDA approval in 2004 for HIV-related facial lipoatrophy, as well as for cosmetic indications under the brand name Sculptra, PLLA has emerged as one of the most widely used fillers [1]. In contrast to hyaluronic acid (HA)-based fillers, which provide immediate volume restoration, the mechanism of action of PLLA relies on fibroblastic stimulation and neo-collagenesis, resulting in gradual volumization of the skin tissue over several months [2,3,4].

The filler is supplied in the form of a lyophilized powder that must be diluted with sterile water before injection. Early approaches used only 2-3 mL of sterile water for dilution, which was associated with a very high rate of nodule formation, according to earlier studies [3]. Subsequent improvements in dilution practices, injection techniques, and post-treatment massage regimens have significantly enhanced the product's safety profile [4,5,6]. Nonetheless, the complications of greatest clinical concern are delayed nodules and granulomatous reactions, which account for 8.3% of all filler-related adverse events [7].

One major difficulty in dealing with PLLA nodules stems from the fact that these lesions may arise after an extended period, with a wide clinical presentation and a variety of pathological processes, ranging from the formation of microscopic particles and type IV hypersensitivity granulomatous reactions to, in rare cases, bacterial infection, most commonly involving Staphylococcus epidermidis biofilm formation [8,9]. This study aims to bridge this knowledge gap via conducting a PRISMA-ScR (Preferred Reporting Items for Systematic Reviews and Meta-Analyses extension for Scoping Reviews)-compliant scoping review of the current scientific literature regarding the development, diagnostics, classification, and treatment of PLLA nodules, while simultaneously creating a clinically viable pathway for dealing with the issue based on evidence-based guidelines.

## Review

Search strategy and study selection

A thorough search was conducted in four databases, namely PubMed/MEDLINE, Scopus, Web of Science (Core Collection), and Cochrane Library. The search process was conducted using a Boolean search strategy that used key terms for PLLA, Sculptra, fillers, nodules, papules, granulomas, adverse reactions, and their diagnosis and management. Searches were limited to articles published in the English language from 2000 up to April 2026. No gray literature, personal opinions, non-peer-reviewed publications, or non-human studies were included.

The criteria for patient, concept, and context (PCC) were used to screen the eligibility of the study. The patient refers to people who developed nodules, papules, granulomas, and localized adverse reactions following the injection of PLLA. The context entailed all scenarios where PLLA was being applied in a clinical setting. While the main emphasis of this scoping review was on PLLA-related nodule formation and localized delayed adverse events, a few articles were still included despite reporting significant vascular and visual complications associated with PLLA injections because of their importance in the identification and differential diagnosis of cosmetic filler-related complications. Reported vascular complications included vascular occlusion, tissue ischemia, skin necrosis, and embolic events, whereas visual complications included retinal artery occlusion, visual impairment, and, in rare cases, permanent vision loss.

**Figure 1:**
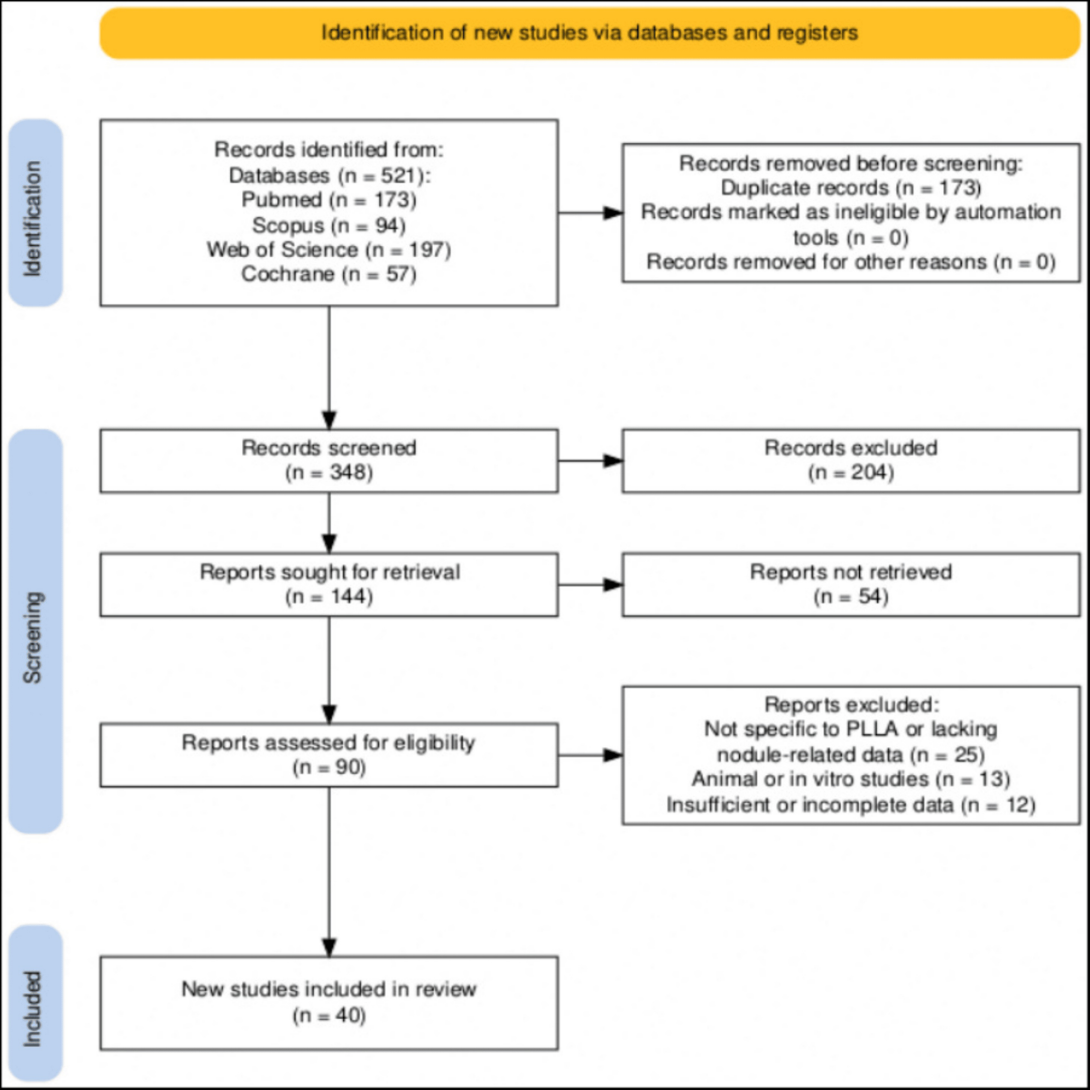
PRISMA-ScR flow diagram: management of PLLA-associated nodules: scoping review PRISMA-ScR: Preferred Reporting Items for Systematic Reviews and Meta-Analyses extension for Scoping Reviews; PLLA: poly-L-lactic acid

Database searches retrieved 521 references (PubMed = 173, Scopus = 94, Web of Science = 197, and Cochrane = 57). Following de-duplication using a three-stage process involving DOI identification, author-year-journal similarity checks, and fuzzy title matching, 348 unique records remained for title and abstract screening. After excluding 204 records during screening, 144 reports were sought for retrieval. Of these, 54 reports could not be retrieved, leaving 90 reports assessed for eligibility. Subsequently, 50 reports were excluded for the following reasons: lack of PLLA-specific or nodule-related data (n = 25), animal or in vitro studies (n = 13), and insufficient or incomplete data (n = 12). Ultimately, 40 studies met the inclusion criteria and were included in the final scoping review. The study selection process is illustrated in Figure 1.

Quality appraisal

Quality appraisal was done to assess the methodological quality and level of evidence in the included studies and to facilitate the development of clinical pathways. While scoping reviews do not necessitate a mandated critical appraisal as per the JBI Manual for Evidence Synthesis, a critical appraisal was done to weight evidence and highlight methodological weaknesses. Various design-specific Joanna Briggs Institute (JBI) Critical Appraisal Tools were used, including the case report tool, case series tool, cohort studies tool, randomized control trial (RCT) tool, systematic review tool, cross-sectional study tool, and text opinion paper tool.

Among the 40 studies, 14 were case reports, 12 were case series, and six were retrospective cohort or chart review studies, whereas other studies included RCTs, systematic reviews, prospective cohort studies, expert consensus statements, and mixed-method studies. All studies were appraised in terms of eight domains encompassing methodology, measures of outcomes, confounders, follow-ups, and completeness of reporting. Studies received ratings as excellent (8/8), good (6-7/8), fair (4-5/8), or poor (<4/8). Overall, most studies were found to have methodological quality that ranged from fair to excellent (Table 1).

**Table 1:** Quality appraisal Quality grades: excellent (8/8 criteria met), good (6-7/8), fair (4-5/8), poor (<4/8). Evidence strength classified per JBI guidance: high (RCT/SR), moderate-high (prospective cohort/registry), moderate (retrospective/expert consensus), low-moderate (case series), low (case report/expert opinion) JBI: Joanna Briggs Institute; Y: yes; n: no; u: unclear; N/A: not applicable

Author (year)	Study design	JBI tool used	Q1: Patient sampling/randomization	Q2: Exposure/condition clearly defined	Q3: Outcomes measured reliably	Q4: Appropriate statistical analysis/case description	Q5: Confounders identified	Q6: Follow-up adequate	Q7: Outcomes reported completely	Q8: Appraisal item 8	Total score	% Quality	Quality grade
Stewart et al. (2007) [10]	Case series	JBI CS	Y	Y	Y	Y	U	Y	Y	Y	7	87%	Good
Apikian et al. (2007) [11]	Case series	JBI CS	Y	Y	Y	Y	N	Y	Y	Y	7	87%	Good
Alijotas-Reig et al. (2009) [12]	Case series	JBI CS	Y	Y	Y	Y	U	Y	Y	Y	7	87%	Good
Reszko et al. (2009) [13]	Case report	JBI CR	Y	Y	Y	Y	N/A	Y	Y	Y	7	88%	Good
O'Daniel (2017) [14]	Case series	JBI CS	Y	Y	Y	Y	N	Y	Y	Y	7	87%	Good
Lee et al. (2017) [15]	Case report	JBI CR	Y	Y	Y	Y	N/A	Y	Y	Y	7	88%	Good
Jeon et al. (2020) [16]	Case report	JBI CR	Y	Y	Y	Y	N/A	Y	Y	U	6	75%	Fair
Vrcek et al. (2017) [17]	Case report	JBI CR	Y	Y	Y	Y	N/A	Y	Y	U	6	75%	Fair
Roberts and Arthurs (2012) [1]	Case report	JBI CR	Y	Y	Y	Y	N/A	Y	Y	Y	7	88%	Good
Wu and Goldman (2016) [5]	RCT	JBI RCT	Y	Y	Y	Y	Y	Y	Y	Y	8	100%	Excellent
Vleggaar et al. (2014) [2]	Expert consensus	JBI Text/Opinion	Y	Y	Y	Y	U	Y	Y	Y	7	87%	Good
Narins (2008) [3]	Expert opinion/review	JBI Text/Opinion	Y	Y	U	Y	U	Y	Y	U	5	63%	Fair
Fiore et al. (2013) [18]	Case report	JBI CR	Y	Y	Y	Y	N/A	Y	Y	Y	7	88%	Good
Ianhez et al. (2024) [19]	Retrospective cohort	JBI CO	Y	Y	Y	Y	Y	Y	Y	Y	8	100%	Excellent
Wortsman et al. (2025) [20]	Cross-sectional	JBI Cross-sectional	Y	Y	Y	Y	Y	Y	Y	Y	8	100%	Excellent
Kern et al. (2022) [7]	Retrospective cohort	JBI CO	Y	Y	Y	Y	Y	Y	Y	Y	8	100%	Excellent
Palm et al. (2021) [6]	Retrospective chart review	JBI CO	Y	Y	Y	Y	U	Y	Y	Y	7	87%	Good
Lowe et al. (2009) [21]	Case series	JBI CS	Y	Y	Y	Y	U	Y	Y	Y	7	87%	Good
Palm et al. (2010) [22]	Retrospective cohort	JBI CO	Y	Y	Y	Y	U	Y	Y	Y	7	87%	Good
Vleggaar et al. (2014b) [4]	Expert consensus	JBI Text/Opinion	Y	Y	Y	Y	Y	Y	Y	Y	8	100%	Excellent
Guardiani and Davison (2012) [23]	Case report	JBI CR	Y	Y	Y	Y	N/A	Y	Y	Y	7	88%	Good
Alijotas-Reig et al. (2013) [8]	Systematic review	JBI SR	Y	Y	Y	Y	Y	Y	Y	Y	8	100%	Excellent
Lee and Kim (2015) [24]	Review	JBI Text/Opinion	Y	Y	Y	Y	U	Y	Y	Y	7	87%	Good
Kates and Fitzgerald (2008) [25]	Case series with review	JBI CS	Y	Y	Y	Y	U	Y	Y	Y	7	87%	Good
Amselem et al. (2024) [26]	Prospective cohort	JBI CO	Y	Y	Y	Y	Y	Y	Y	Y	8	100%	Excellent
Vasconcelos-Berg et al. (2024) [27]	Retrospective cohort	JBI CO	Y	Y	Y	Y	Y	Y	Y	Y	8	100%	Excellent
Wang et al. (2025) [28]	Systematic review	JBI SR	Y	Y	Y	Y	Y	Y	Y	Y	8	100%	Excellent
Choi et al. (2024) [29]	Case report	JBI CR	Y	Y	Y	Y	N/A	Y	Y	U	6	75%	Fair
Marinho et al. (2025) [30]	Case report	JBI CR	Y	Y	Y	Y	N/A	Y	Y	Y	7	88%	Good
Cortez et al. (2024) [31]	Case report	JBI CR	Y	Y	Y	Y	N/A	Y	Y	U	6	75%	Fair
Cao et al. (2025) [32]	Clinical trial	JBI RCT	Y	Y	Y	Y	Y	Y	Y	Y	8	100%	Excellent
Tetzner et al. (2025) [33]	Case series	JBI CS	Y	Y	Y	Y	U	Y	Y	Y	7	87%	Good
Haneke (2019) [34]	Review	JBI Text/Opinion	Y	Y	U	Y	U	Y	Y	U	5	63%	Fair
Khorasanizadeh et al. (2026) [35]	Review	JBI Text/Opinion	Y	Y	Y	Y	U	Y	Y	Y	7	87%	Good
Xu et al. (2024) [36]	Systematic review	JBI SR	Y	Y	Y	Y	Y	Y	Y	Y	8	100%	Excellent
Saththianathan et al. (2017) [9]	Mixed methods	JBI CO	Y	Y	Y	Y	Y	Y	Y	Y	8	100%	Excellent
Rendon (2012) [37]	Case series	JBI CS	Y	Y	Y	Y	U	Y	Y	Y	7	87%	Good
Goldman (2011) [38]	Case series/review	JBI CS	Y	Y	U	Y	N	Y	Y	U	5	63%	Fair
Wu and Wu (2021) [39]	Case report	JBI CR	Y	Y	Y	Y	N/A	Y	Y	Y	7	88%	Good
Burgess and Quiroga (2005) [40]	Prospective case series	JBI CS	Y	Y	Y	Y	U	Y	Y	Y	7	87%	Good

Study characteristics

Of the 40 included articles, 14 case reports, 12 case series, six retrospective cohorts/chart review studies, two RCTs, four systematic reviews/meta-analyses, and two consensus opinions were identified. Details of all the included articles are listed in Table 2.

**Table 2:** Characteristics of included studies (n = 40) PLLA: poly-L-lactic acid; IL: intralesional; HFUS: high-frequency ultrasound; RCT: randomized controlled trial; NTM: non-tuberculous mycobacteria; PDLLA: poly-D,L-lactic acid; 5-FU: 5-fluorouracil; HIV: human immunodeficiency virus

Author (year)	Country	Study design	Key findings
Stewart et al. (2007) [10]	USA	Case series (n = 10)	Periorbital PLLA granulomas; IL steroids effective; surgical excision 1/10
Apikian et al. (2007) [11]	Australia	Case series (n = 4)	Periorbital delayed nodules; triamcinolone + 5-FU; partial resolution
Alijotas-Reig et al. (2009) [12]	Spain	Case series (n = 13)	Immune-mediated nodules; mean onset 12 mo; recurrence 31%; hydroxychloroquine used
Reszko et al. (2009) [13]	USA	Case report	Late nodule 18 mo post-injection; MRI; resolved with IL triamcinolone
O'Daniel (2017) [14]	USA	Case series (n = 7)	Recurrent nodules 6–24 mo; excision 3/7; recurrence linked to low dilution
Lee et al. (2017) [15]	Taiwan	Case report	Foreign body granuloma at 24 mo; biopsy confirmed; resolved with IL steroids
Jeon et al. (2020) [16]	South Korea	Case report	Late-onset nodule 18 mo; histology confirmed; conservative resolution
Vrcek et al. (2017) [17]	USA	Case report	Neuropathic pain post-PLLA; MDT approach; partial resolution
Roberts and Arthurs (2012) [1]	Canada	Case report	Visual loss + orbital infarction periorbital PLLA; irreversible harm
Wu and Goldman (2016) [5]	USA	RCT (n = 50)	Massage alone insufficient; dilution more critical for nodule prevention
Vleggaar et al. (2014) [2]	Multi-country	Expert consensus	Comprehensive adverse event framework; management ladder
Narins (2008) [3]	USA	Expert opinion	≥6 mL dilution; ≥2 hr reconstitution; massage protocol
Fiore et al. (2013) [18]	USA	Case report	M. mucogenicum infection post-PLLA; culture before steroids critical
Ianhez et al. (2024) [19]	Brazil	Retrospective cohort (n = 55)	55 cases; nodules 43.6%; resolution 85%; technique correction paramount
Wortsman et al. (2025) [20]	Multi-country	Cross-sectional (n = 118)	HFUS characterizes PLLA granulomas; layer-depth guided treatment
Kern et al. (2022) [7]	USA	Retrospective cohort (n = 7,659)	PLLA 8.3% of serious events; delayed nodules most common
Palm et al. (2021) [6]	USA	Chart review (n = 150)	10–12 mL dilution: nodule rate 1.3% vs 8% standard protocol
Lowe et al. (2009) [21]	USA/UK	Case series (n = 100)	Nodule rate 4% with ≥6 mL; 96% resolved conservatively
Palm et al. (2010) [22]	USA	Retrospective cohort (n = 130)	8% nodule rate; 92% resolved with massage/steroids
Vleggaar et al. (2014b) [4]	Multi-country	Expert consensus	Gold-standard 3-step management ladder; 8–10 mL dilution
Guardiani and Davison (2012) [23]	USA	Case report	Angioedema 6 wk post-PLLA; resolved with antihistamines + IV steroid
Alijotas-Reig et al. (2013) [8]	Spain	Systematic review	Type IV hypersensitivity; immunomodulation for immune-mediated nodules
Lee and Kim (2015) [24]	South Korea	Review	Foreign body granuloma pathophysiology; treatment hierarchy
Kates and Fitzgerald (2008) [25]	USA	Case series + review (n = 12)	HIV population; higher nodule risk; conservative management 85%
Amselem et al. (2024) [26]	Europe	Prospective cohort (n = 98)	Lanluma PLLA; nodule rate 2%; high-dilution 10–16 mL
Vasconcelos-Berg et al. (2024) [27]	Brazil	Retrospective cohort (n = 250)	Immediate reconstitution safe; nodule rate 3.2%; dilution 8–12 mL
Wang et al. (2025) [28]	China	Systematic review	Type IV immunological granuloma confirmed; management hierarchy
Choi et al. (2024) [29]	South Korea	Case report	PDLLA eyelid granuloma; IL triamcinolone; complete resolution
Marinho et al. (2025) [30]	Brazil	Case report (2 cases)	Elleva PLLA refractory nodules; excision required; hyaluronidase ineffective
Cortez et al. (2024) [31]	Brazil	Case report	PLLA-induced scalp alopecia; perifollicular granuloma; largely irreversible
Cao et al. (2025) [32]	China	Clinical trial (n = 82)	Porous PLLA microspheres: nodule rate 1.2% vs 8.9% standard
Tetzner et al. (2025) [33]	Brazil	Case series (n = 28)	Oral/maxillofacial PLLA granulomas; 71% resolved; early specialist referral
Haneke (2019) [34]	Germany	Review	Adverse effects classification: early papules vs late granulomas
Khorasanizadeh et al. (2026) [35]	Iran/Multi	Review	HFUS patterns of PLLA nodules; depth-guided treatment planning
Xu et al. (2024) [36]	China	Systematic review	Nodule risk 2–14%; dilution/reconstitution/depth key preventive factors
Saththianathan et al. (2017) [9]	Australia	Mixed methods (n = 38)	Biofilm 62% of filler complications; antibiotics improve outcomes to 88%
Rendon (2012) [37]	USA	Case series (5-year)	Long-term safety confirmed; 95%+ resolution with conservative management
Goldman (2011) [38]	USA	Case series/review	'5-5-5 rule': 5 mL, 5 min, 5 days; IL steroids for established nodules
Wu and Wu (2021) [39]	Taiwan	Case report	Retinal artery occlusion post-temporal PLLA; irreversible vision loss
Burgess and Quiroga (2005) [40]	USA	Prospective case series (n = 28)	HIV lipoatrophy; nodule rate 4% at 48 wk; manageable conservative measures

Clinical presentation and time of onset

The presentations of PLLA-associated nodules can differ widely with respect to their seriousness, morphologic features, symptoms, and time of occurrence. The most common presentation is that of firm subcutaneous nodules or papules that are either solitary or multiple and vary in size from less than 5 mm up to more than 10 mm. The differences in the incidence rates between various research studies are mainly attributable to variations in the techniques of administration, methods of dilution, study follow-up periods, populations of patients, and definitions of palpable nodules, micronodules, and granulomatous reactions.

In their work, Palm et al. documented the incidence of nodules among 130 subjects as 11 out of 130 patients, with the incidence rate being 8.5%. The rates among individual facial sites included cheek, 7.2%; temporal, 4.2%; nasolabial/marionette, 3.9%; and hand, 12.5% [22]. On the other hand, Alijotas-Reig et al. documented granulomatous reaction rates that varied from 0.1% to 12% in past reports in the literature, but this estimate also included other clinical entities such as palpable nodules and micronodules [12]. Incidence figures must thus be interpreted with caution since many studies are case series and have no denominators provided.

In addition, post-marketing surveillance studies have shown that nodules can occur as one of the complications resulting from Sculptra treatments. Sculptra: As per Rayess HM et al., after analyzing data from the United States FDA’s Manufacturer and User Facility Device Experience (MAUDE) database, there were 17 cases of nodules, which accounted for 36.1% of all Sculptra complications reported in their study. The incidence rate was found to be about 0.004% of all Sculptra procedures carried out, as per data collected by the American Society of Plastic Surgeons. It was also highlighted by the researchers that MAUDE is a voluntary database and hence prone to underreporting and reporting bias [41,42].

PLLA-induced nodules can be categorized as either early or delayed onset. Early onset nodules, which occur in the first few weeks following injection, are mostly non-inflammatory and are said to be due to aggregation of PLLA particles or early host response [2,3,34]. On the other hand, delayed onset nodules, which occur several months after the treatment, suggest a foreign body granulomatous reaction to PLLA implants [8,12,15]. Bimodal categorization appears to be more practical, as distinguishing intermediate onset nodules can be difficult in clinical settings.

The mean time of presentation for delayed onset nodules was between nine and 14 months post-procedure, although some reported instances showed cases beyond 24 months [10,15]. Unusual presentations that occurred according to the current literature review were neuropathic pain due to lack of clinical evidence of a lesion [17], diffuse facial edema [23], and hair loss from alopecia due to perifollicular granuloma from improper scalp injection [30]. Less frequently observed side effects involved vasculopathic conditions and visual impairment, such as retinal artery occlusion with permanent visual impairment [1,39]. While not the primary concern of this review, these less-common complications were still considered due to their potential importance within the context of complication identification, patient safety, and differential diagnosis in aesthetic procedures.

Diagnostic approach and differential diagnosis

Diagnosis of PLLA-related nodules is dependent on clinical history taking that includes an evaluation of the previous injection procedure with respect to the type of filler material used, dilution volume, time of reconstitution, depth of injection, injection technique, and number of treatments [2,24,34]. Physical examination should focus on lesion size, consistency, tenderness, erythema, skin integrity, anatomic distribution, and onset time. Differing from PLLA-related nodules, the differential diagnosis should include other fillers such as HA, CaHA, PDLLA, PCL, epidermoid cysts, inclusion cysts, lipomas, lymphadenopathy, bacterial abscesses, sarcoidosis, granulomatous diseases, and, rarely, neoplasms [2,24,34]. Nodules caused by HA are distinct in their potential for responding to hyaluronidase treatment.

Conventional ultrasound (US) can be applied as a useful first-line imaging modality in cases of suspected PLLA-related nodules, especially those that are palpable or extend deep into subcutaneous fat layers. Conventional US can be employed in localizing the filler deposits, evaluating edema or soft tissue masses, determining the size and depth of the lesion, and determining the presence of vasculature in color Doppler ultrasound. However, the limitation of conventional ultrasound is its inability to detect superficial or small PLLA deposits owing to poor spatial resolution [24,34].

HFUS, with probe frequencies ranging from 15 to 22 MHz, is highly sensitive in the diagnosis of PLLA-related lesions. In one of the largest multicenter imaging studies performed in 118 patients, characteristic hypoechoic changes have been described with well-defined parameters dependent on lesion depth, size, and distribution [20]. Moreover, HFUS helps differentiate PLLA injections from other dermal filler types and can help guide intralesional treatments without inappropriate needle placements. Successful results have been achieved in almost 84% of cases with HFUS-guided therapy [20].

Biopsy and histopathology remain the gold standards in establishing a diagnosis of granuloma formation, especially if the lesion is persistent, inflamed, unusual, or inconclusive [8,15,24]. Histopathologically, typical foreign body granulomas consist of multinucleated giant cells containing birefringent PLLA particles in a background of chronic inflammation [8,15,24].

In immune-related conditions, immunohistopathology has shown dominance of infiltrate of T-cells identified as CD3+ and CD4+. Before giving steroids or immunosuppression in suspected infections, it is necessary to obtain a culture because of possible steroid-induced infection caused by atypical mycobacteria such as Mycobacterium mucogenicum after PLLA injection [18].

MRI was performed in a selected number of cases where there was deep-tissue involvement, peri-orbital involvement, neural involvement, or where malignancies needed to be ruled out [13,17]. While MRI is not always necessary in PLLA nodule diagnosis, it can be helpful in complicated diagnoses. A suggested diagnostic classification and differential diagnosis scheme is presented in Table 3.

**Table 3:** Clinical classification and diagnostic framework for PLLA-associated nodules PLLA: poly-L-lactic acid; HA: hyaluronic acid; HFUS: high-frequency ultrasound; US: ultrasound; CT: computed tomography; MRI: magnetic resonance imaging; NTM: non-tuberculous mycobacteria

Nodule type	Clinical features	Typical onset	Recommended diagnostic approach	Key differentials to exclude
Type A: non-inflammatory nodule	Firm, palpable, non-tender nodules without erythema or systemic manifestations	Early (4 weeks–6 months) or delayed (>6 months)	Clinical examination; US/HFUS if diagnosis is uncertain	Epidermoid cyst, lipoma, normal anatomical structures, HA filler nodule
Type B: inflammatory nodule	Tender, erythematous, indurated lesions with or without inflammatory symptoms	Typically delayed (>6 months); variable onset	Clinical examination with HFUS; biopsy/histopathology when persistent or atypical	HA granuloma, bacterial abscess, sarcoidosis, granulomatous disease, lymphoma
Type C: infectious nodule	Tender, warm swelling with possible purulent discharge, erythema, or fever; poor response to steroids	Variable; commonly within weeks after injection	Microbiological culture before corticosteroids; US/HFUS; CT if deep extension or systemic spread suspected	Bacterial abscess, PLLA granuloma, herpes zoster, atypical mycobacterial infection
Type D: atypical presentation	Neuropathic pain, diffuse angioedema, alopecia, or non-specific symptoms without discrete palpable nodules	Variable; early or delayed	Multidisciplinary assessment; targeted imaging (MRI/US/HFUS); neurological or dermatological evaluation when indicated	Angioedema, neuropathy, scarring alopecia, systemic hypersensitivity reactions

Management strategies and therapeutic options

Prevention of PLLA Nodule Formation

Prevention continues to represent the most evidence-based method of nodule prevention related to the administration of PLLA. Based on current scientific evidence, appropriate dilution, correct reconstitution, proper injection depth, and non-bolus delivery represent the most important modifiable aspects. In a study conducted by Wu & Goldman, the authors demonstrated that only massage without appropriate dilution and injection techniques could not be sufficient for nodule prevention [5]. Thus, the volume of dilution should play a critical role as a preventive factor compared to massage only.

Evidence provided by cohorts and expert consensus clearly demonstrates the need to increase the volume of the dilution up to 8-12 ml of sterile water per vial, with even higher amounts recommended in some cases based on the size of the treatment region [2-4,6,27]. Expert consensus also recommends adequate reconstitution time, which usually starts from two hours, with even longer periods of time recommended when possible [2-4]. Although post-injection massage, such as the “5-5-5 rule,” is a common practice, there are no robust randomized studies demonstrating its preventive impact independently [5,38].

Adjunctive Imaging and Image-Guided Interventions

In the case of mild, non-inflammatory PLLA nodules, an option could involve watchful waiting, patient counseling, and massage therapy, especially in cases where the lesion size is small, non-tender, and cosmetically insignificant [2,5]. Since the rates of spontaneous resolution among the available studies differ widely in their methodologies and are not validated by denominator-based studies, estimates on percentages of lesion resolution should be carefully avoided, unless specifically indicated in the cited literature.

Nodules classified as moderate to severe would require intralesional corticosteroids, which usually involve injection with triamcinolone acetonide, especially when the lesions are palpable, inflamed, and cosmetically significant [2,19,28]. The use of ultrasound or high-frequency ultrasound should be considered in the treatment of deep-seated or cosmetically sensitive lesions [20].

Other alternatives for recurrent, severe, and inflammatory nodules are intralesional 5-fluorouracil alone or in association with triamcinolone as outlined by the expert consensus and reports based on specific cases [2,4,24]. For immune-mediated granulomatous reactions, systemic or immunomodulating therapy using corticosteroids or hydroxychloroquine could be applied in a number of cases. These measures must be tailored according to each patient [8,12].

The presence of infection or biofilm may be considered in cases of painful, erythematous, recurrent, and draining nodules or those that are resistant to corticosteroid treatment. Cultures should always precede corticosteroid treatment or immunosuppressants. The use of antibiotics may be necessary if infections or biofilms are suspected. Treatments should be based on clinical evidence and cultures [9,18].

The option of surgery should be used as a last resort in patients with persistent or fibrotic nodules that do not respond to any other form of management [14,30,33]. Localization of the lesion with ultrasonography or high-frequency ultrasound might prove useful in some cases. Complications of a vascular or visual nature mentioned in rare case studies were not included in the nodular treatment algorithm, since they do not pertain to the scope of the review under discussion; nevertheless, they emphasize the significance of anatomical awareness, proper injection techniques, and immediate specialist consultation in case of vascular complications [1,39]. The hierarchical management scheme is presented in Table 4.

**Table 4:** Evidence-mapped management algorithm for PLLA-associated nodules PLLA: poly-L-lactic acid; HA: hyaluronic acid

Severity grade	Clinical criteria	Recommended treatment	Expected outcomes	Evidence level
Grade 1: mild	Single, non-tender palpable nodule; <5 mm; no skin change or functional impairment	Reassurance, massage, warm compresses, and short-term clinical observation	Many mild nodules may improve spontaneously or remain clinically stable with conservative management	Low
Grade 2: moderate	Multiple or persistent nodules; mild tenderness or erythema; approximately 5–10 mm; cosmetic concern	Intralesional triamcinolone acetonide (commonly 5–10 mg/mL); repeat sessions may be considered according to clinical response; US/HFUS guidance may improve localization in selected cases [20,35]	Frequently reported improvement in inflammatory and persistent nodules in observational studies	Moderate
Grade 3: severe	Inflammatory nodules or plaques; >10 mm; tenderness, erythema, or inflammatory symptoms; possible systemic manifestations	Combination therapy including intralesional corticosteroids, intralesional 5-fluorouracil, systemic corticosteroids, or selected immunomodulatory agents such as hydroxychloroquine in immune-mediated cases [8,12,34]	Variable response depending on inflammatory severity, chronicity, and underlying mechanism	Moderate
Grade 4: refractory/complex	Persistent or treatment-resistant nodules; atypical, fibrotic, recurrent, or diagnostically uncertain lesions	Multidisciplinary assessment; surgical excision for refractory lesions; culture-directed antimicrobial therapy when infection or biofilm is suspected [9,14,30,33]	Surgical management may be effective in carefully selected refractory cases	Moderate
Special category: infectious/biofilm-associated	Tender, warm, fluctuant, draining, or culture-positive nodules; poor response to corticosteroids	Microbiological culture before steroids; targeted antibiotic therapy based on suspected organism and sensitivity testing; drainage if fluctuant. Reported regimens include clarithromycin, doxycycline, minocycline, ciprofloxacin, rifampicin combinations, and anti-staphylococcal coverage where appropriate [9,18]	Improvement may occur with early culture-directed antimicrobial management	Moderate

Follow-Up and Recurrence

It is advised that long-term follow-up be considered for those who have been undergoing treatment for PLLA nodules due to their potential for late inflammatory response and repeated occurrence, which have been observed in some research studies and case series [9,12,28]. Unfortunately, pooled estimates regarding recurrence risk are not yet available owing to the fact that most previous research done was on individual cases with heterogeneous groups and varied follow-up approaches [12,14]. Recurrence has often been observed among inflammatory/immune nodules and poor techniques used in dilution/injection [12,14]. Some possible causes of recurrence mentioned are infection, dental work, and immunosuppressive events [12].

According to the literature review, follow-up can involve re-evaluation of the patient four weeks after the first intralesional administration if a partial response has occurred, and then further evaluation of the condition at around three, six, and 12 months to track lesion healing, recurrence, cosmetic results, and adverse effects from treatment. Photographs taken regularly during clinical follow-up might prove helpful for documentation and evaluation purposes. The long-term follow-up studies conducted with patients with prolonged follow-up have reported good results and minimal adverse effects after successful PLLA treatment [37].

Evidence Strength and Evidence Gaps

The scientific literature for managing PLLA nodules is mostly observational. Evidence is highest for the dilution optimization technique as a preventive approach [5,6,32] and for the use of antibiotics as a combined therapy in biofilm nodules [9]. There are plenty of observational studies and consensus supporting intralesional triamcinolone as the first-line therapy, but no randomized controlled trial assessing its efficacy specifically for PLLA nodules has been conducted yet. Other second-line medications include 5-FU and hydroxychloroquine, which have been recommended based on studies on filler granulomas and extrapolation from their results (Table 5). 

**Table 5:** Evidence strength summary by clinical domain IL: intralesional; 5-FU: 5-fluorouracil; HFUS: high-frequency ultrasound; PLLA: poly-L-lactic acid; RCT: randomized controlled trial

Clinical domain	Key supporting studies	Evidence Level	Summary/caveats
Prevention (dilution ≥8 mL)	Wu and Goldman (2016) [5], Palm et al. (2021) [6], Vasconcelos-Berg et al. (2024) [27], Vleggaar et al. (2014) [2], Vleggaar et al. (2014b) [4]	Moderate-High	Higher dilution protocols, commonly ≥8–10 mL depending on product and site, were consistently associated with lower nodule risk compared with lower-volume protocols. Evidence includes RCT, cohort, and consensus data
Prevention (reconstitution 24–48 hrs)	Vleggaar et al. (2014) [2], Narins (2008) [3], Vleggaar et al. (2014b) [4]	Low-Moderate	Supported mainly by expert consensus. Longer reconstitution, commonly 24–48 hours, where recommended, may improve particle dispersion; however, no RCT directly compares reconstitution times
Prevention (massage protocol)	Wu and Goldman (2016) [5], Goldman (2011) [38]	Low-Moderate	Massage is widely recommended, but available evidence suggests it should not be considered sufficient without appropriate dilution and injection technique
HFUS for diagnosis	Wortsman et al. (2025) [20], Khorasanizadeh et al. (2026) [35]	Moderate-High	US/HFUS helps localize filler deposits, assess lesion depth and distribution, distinguish filler-related nodules from other soft tissue lesions, and guide targeted treatment
Biopsy for confirmation	Alijotas-Reig et al. (2009) [12], Lee et al. (2017) [15], Fiore et al. (2013) [18], Alijotas-Reig et al. (2013) [8], Xu et al. (2024) [36]	Moderate	Histopathology remains the confirmatory approach for persistent, inflammatory, atypical, or diagnostically uncertain nodules
IL triamcinolone (first-line)	Stewart et al. (2007) [10], Apikian et al. (2007) [11], Palm et al. (2010) [22], Vleggaar et al. (2014) [2], Vleggaar et al. (2014b) [4]	Moderate	Frequently reported first-line treatment for inflammatory or persistent nodules, but no PLLA-specific RCT has evaluated its efficacy. Avoid exact response percentages unless directly reported by individual studies
IL 5-FU (second-line)	Stewart et al. (2007) [10], Vleggaar et al. (2014) [2], Haneke (2019) [34]	Low-Moderate	Used as a second-line option for persistent or inflammatory filler-related nodules, usually based on limited case-based evidence and extrapolation from broader granuloma management
Hydroxychloroquine	Alijotas-Reig et al. (2009) [12]	Low-Moderate	Reported in immune-mediated or recurrent granulomatous reactions, but evidence is limited to case-series level data and should be individualized
Antibiotic therapy (biofilm)	Saththianathan et al. (2017) [9], Fiore et al. (2013) [18]	Moderate	Antibiotics may be considered when biofilm or infection is suspected, particularly in recurrent, painful, draining, or steroid-nonresponsive nodules. Culture should be obtained before steroids, where infection is possible
Surgical excision	O'Daniel (2017) [14], Marinho et al. (2025) [30], Tetzner et al. (2025) [33]	Low-Moderate	Reserved for refractory, fibrotic, persistent, or diagnostically uncertain nodules after conservative and intralesional options fail. Evidence is mainly case-based
Porous PLLA microspheres	Cao et al. (2025) [32]	Moderate-High	Early clinical trial evidence suggests newer PLLA formulations may reduce nodule risk, but larger independent comparative studies are still needed

Evidence-mapped clinical pathway

**Figure 2:**
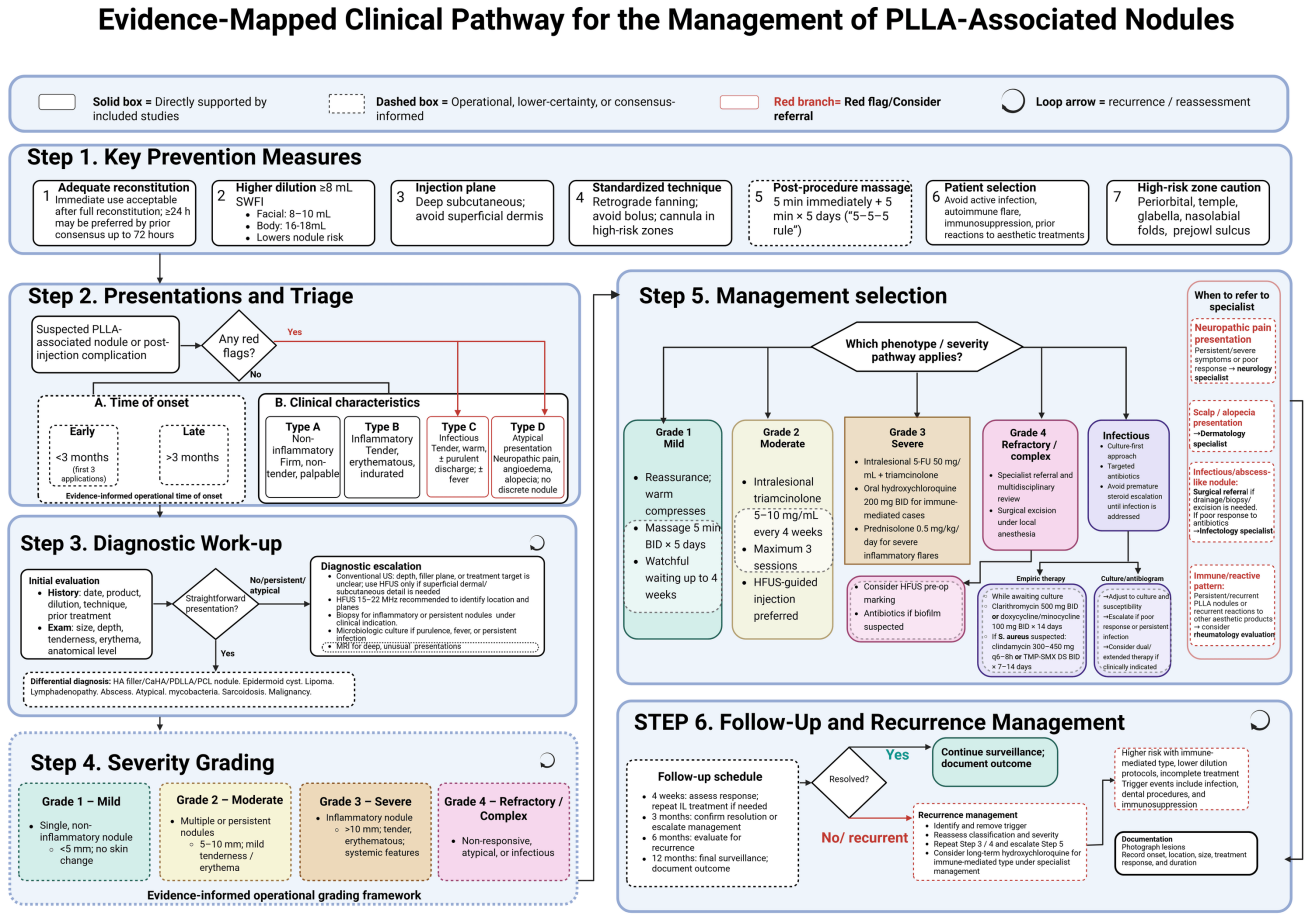
Evidence-mapped clinical pathway for the prevention, diagnosis, severity grading, management, and follow-up of PLLA-associated nodules based on published evidence and expert-informed operational recommendations PLLA: poly-L-lactic acid; SWiFT: sterile water for injection iechnique; 5-FU: 5-fluorouracil; BD: benzoyl peroxide; IFU: instructions for use; US: ultrasound; PO: oral administration; IL: intralesional; CT: computed tomography; MRI: magnetic resonance imaging; PET: positron emission tomography; PCR: polymerase chain reaction; AFB: acid-fast bacilli; NTM: non-tuberculous mycobacteria; FUO: fever of unknown origin; FDA: Food and Drug Administration Created in BioRender. Flores Rodríguez, J. C. (2026) https://BioRender.com/5oiw04q

The evidence-mapped clinical pathway with six stages derived from this scoping review is shown in Figure 2 below. It gives a structured approach to the prevention, detection, classification, treatment, and follow-up of nodules linked to PLLA.

Step 1: Prevention

Correct reconstitution, dilution, injection technique, aseptic handling, and patient selection remain the most significant preventative strategies for PLLA-induced nodules. Appropriate sterile procedures should be followed during reconstitution and handling practices, and the skin area intended for treatment should be free of makeup and undergo enough antisepsis to decrease the potential for introduction of contaminants. Scientific evidence and professional opinion strongly encourage reconstitution of PLLA at least 24 hours before delivery, and numerous writers have recommended 24 to 48 hours to ensure optimal particle hydration. However, there are additional studies that have shown the possibility of immediate administration after reconstitution when a homogeneous, very diluted suspension is successfully created [2-6,27, 38]. 

Higher quantities of diluent have been correlated with decreased rates of nodule formation. Typical dilution methods include about 8-10 mL per vial for facial applications and 16-18 mL or higher for body parts. Deep subcutaneous planes are the preferred site for PLLA injection with retrograde fanning and tunneling procedures. Postprocedure treatment typically includes massage using the “5-5-5” guideline (five minutes, five times a day, for five days); however, there is limited evidence for its independent benefit. Use increased caution in highly mobile or highly anatomically risky areas, such as the periorbital region, temporal area, glabella, nasolabial folds, and prejowl sulcus. In these sites, precise knowledge of regional anatomy and meticulous injection technique are important to decrease the risk of problems [2,4-6,38].

Step 2: Appearance and Triage

PLLA-associated nodules should be categorized by both time of onset and clinical appearance. Literature describes a bimodal pattern of onset with early reactions typically occurring at four to eight weeks and delayed reactions occurring after six months, but this pathway uses a simplified practical surveillance framework using early (<3 months) and late (>3 months) onset categories for clinical decision-making [2,8,19,34,36]. Early reactions are usually due to particle aggregation or a limited tissue reaction, while late reactions are more indicative of an inflammatory or granulomatous process. Lesions should be classified, in addition to chronology, by their clinical characteristics (Type A-D), which include inflammatory signs, tenderness, palpability, fixation, erythema, neuropathic symptoms, and related aesthetic or functional repercussions. *Step 3: Work-up*

Adequate work-up is important for proper management. Relevant clinical information should comprise the filler product utilized, volume of dilution, reconstitution protocol, depth and technique of injection, onset of symptoms, and previous treatments. Ultrasonography (US) can be the first-line imaging technique for detecting filler deposits, inflammatory alterations, or fluid collections, and it can also assist in treatments. High-frequency ultrasonography (HFUS; 15-22 MHz) [20,35] can provide further help in assessing the depth, shape, extent, and tissue involvement of the lesion. Histopathological testing should only be performed for atypical, chronic, inflammatory, or treatment-resistant nodules. If infection is suspected, a bacterial culture should be performed before the biopsy, if possible. MRI can be considered in selected difficult cases [9,13,17,18,20,35]. *Step 4: Grading* 

Grading severity is vital for the choice of appropriate treatment modalities. Grade 1 (mild) nodules are single, non-inflammatory lesions less than 5 mm in size with negligible aesthetic effect. Grade 2 (moderate) nodules are frequently several or persistent 5-10 mm lesions and may be painful or erythematous. Grade 3 (severe): prominent inflammatory characteristics or lesion size > 10 mm with accompanying systemic symptoms.

Step 5: Nodule Management

Individualization of management is necessary based on nodular grades, inflammation, and hypothesized mechanisms. Grade 1 nodules can be approached conservatively using reassurance, massage, warm compresses, and watchful waiting. Management of grade 2 nodules usually involves intralesional triamcinolone injection at four-week intervals if indicated. Grade 3 nodules caused by inflammation or immunologic etiologies may need combined therapies comprising intralesional corticosteroid, 5-fluorouracil, systemic corticosteroids, or certain immunomodulating drugs like hydroxychloroquine. Bacterial infection-related or biofilm nodules will necessitate identification through microbiological tests, followed by culture-directed antibiotics and drainage. Grade 4 nodules resistant to treatment or complex in nature will need evaluation from different specialists, surgery, and targeted treatments [2,4,8,9,12,14,18,20,28,30].

Step 6: Follow-Up

Long-term follow-ups are indicated to assess therapeutic efficacy, any recurrence of disease, and the cosmetic results. Re-evaluation can be done at about four weeks, three months, six months, and one year after treatment, with serial standardized photos taken at each time point to objectively monitor progress. For recurrent cases, possible reasons such as insufficient dilution, immunologically mediated inflammation, or infections need re-evaluation. Further therapy can be escalated depending on the severity and type of recurrence, with prolonged immunotherapy being one possibility in some recurrent inflammatory cases [2,4,8,12,19,28,36].

Comparative discussion: findings of this scoping review against the broader literature

The 40 included studies are the most concentrated and methodically screened scoping review on PLLA-specific nodule research so far. Putting this research into context with other research on fillers' adverse events helps demonstrate the relevance of this research as well as the limitations of the field.

Some significant studies in the more general filler adverse events literature, which did not include enough specific information on PLLA nodules, were not included in the current scoping review. However, it would be worth comparing them and highlighting some conclusions. For example, Engelhard et al. [42] analyzed the safety data of clinical trials on Sculptra injections and found that the product had a favorable safety profile, with only 1.4% of nodule cases reported in controlled experiments. It contrasts significantly with the results found in the current review, nodule rates from 8 to 14% for the cohorts that used previous, less diluted PLLA solutions of 5 mL. As mentioned previously, one of the limitations is that controlled trial conditions are different from the actual clinic setting.

According to Trinh et al. [43] in their systematic review on delayed complications after tear trough augmentation with dermal fillers, it appears that delayed onset inflammation nodules are more common when using non-HA fillers, but since they do not present results according to the type of filler used in a way that enables isolation of PLLA results, we have excluded the study in our systematic review.

The study carried out by Daines and Williams [44] in a five-year retrospective analysis demonstrated that PLLA accounted for a substantial proportion of dermal filler-related complications, representing 22.4% of all reported complications, although the highest complication rate was observed with calcium hydroxylapatite fillers (41.8%). These findings highlight that, despite being generally considered safe, PLLA remains associated with a clinically relevant burden of adverse events requiring recognition and appropriate management. This is consistent with our finding on PLLA as a cause of serious adverse reactions in one of the largest studies in a single practice [7]. Nevertheless, there was no stratification done for the two parameters; thus, it is impossible to calculate how many events were linked to modifiable technique errors. The current systematic review seeks to bridge the gap, as at least four articles [5,6,27,32] suggest that the protocol modification helps to significantly decrease the rate of nodules, an individual-level analysis that is currently missing from cross-product safety registries.

The article by Bachmann et al. [45] analyzed reactions related to consecutive injection of different filler products in one facial region. It has been found that the prior experience with any HA filler products had no impact on the development of granulomas in PLLA-treated sites, which contradicts previous assumptions about possible sensitization. This conclusion somewhat contradicts the immune-related pathway of reactions discovered through some of the articles reviewed here [12,8], as PLLA appears to be a factor that triggers independent reactions in T cells, regardless of filler products previously used. One possible reason for the disagreement might be associated with the fact that Bachmann et al. performed a 12-month follow-up, while according to our results, late-onset granulomas usually develop within a 12-24 month period [12,15].

In another study that assessed the complications related to fillers reported to the FDA MedWatch system, Ortiz et al. [46] listed PLLA as one of the fillers associated with the reports of granulomas. The authors also noted the lack of a relationship between reporting frequency and incidence estimates, illustrating the inherent weaknesses of the passive monitoring system and the challenge in determining accurate incidence estimates. The existence of such an underestimation problem in systemic monitoring systems underlines the importance of institutional case series and cohorts in the field, precisely what this review focuses on, which explains why there is such variation (1.2% to 14%) in incidence rates throughout studies.

While the consensus document by Avelar et al. [47] specifically targeting Asians and the review article about PLLA in general by Lorenc et al. [48] did not provide enough information about the management of nodules, both recommended higher dilution volumes (≥10 mL) as a standard protocol applicable to all patients, including those with Asian skin types. This finding agrees with the dilution findings summarized by this review [5,6,4,27]. The biofilm mechanism discovered by Saththianathan et al. [9] was not mentioned by either paper, which is a significant lacuna in contemporary international guidelines on the subject.

King et al. suggested an approach to the treatment of delayed onset nodules with signs indicative of biofilm formation or a low-grade infection that are painful, tender, warm, erythematous, or poorly responsive to corticosteroid treatment [49]. Potential treatment options include oral monotherapy with either a macrolide (clarithromycin 500 mg bid) or a tetracycline (doxycycline or minocycline 100 mg bid), followed by reassessment after two weeks. Patients who show a partial response can continue with the same regimen, while those with persisting symptoms need to be treated with combination antibiotics. Regimens suggested include clarithromycin combined with one or more antibiotics like doxycycline, minocycline, or ciprofloxacin. Ciprofloxacin needs to be used with caution due to its side effect profile.

In case of *Staphylococcus* *aureus *infection, anti-staphylococcal antibiotics should be considered. Ideally, treatment should be guided by microbiological culture results and sensitivities. Other drugs reported in the literature include clindamycin, trimethoprim-sulfamethoxazole, tetracyclines, and penicillins in the case of presumed MSSA infections. Additionally, rifampicin was mentioned as an additional treatment option in case of confirmed or presumed staphylococcal biofilms, but it should not be used alone [49].

Some promising adjunctive methods have also been reported in cases of resistant or complex PLLA nodules; however, scientific evidence in this regard is still inadequate and primarily based on experimentation. Radiofrequency using monopolar current has shown its effectiveness against PDLLA nodules in some isolated cases, with possible explanations based on the thermal effect on the porosity and hydratability of the filler; yet, since PDLLA has distinct structural and biological features from PLLA, conclusions cannot be made based on this study alone [50].

High-intensity focused ultrasound (HIFU) therapy has also been utilized in the non-invasive management of non-inflammatory PLLA nodules, albeit in some very isolated case studies with insufficient follow-up periods and minimal histopathological correlation [51]. In another study, a single case study has been reported on the use of immediate radiofrequency after PLLA injection in the absence of nodules at least during short follow-ups [52].

The use of systemic medications should always be approached with caution and employed only in cases of recurring inflammation or immunologically mediated complications. Colchicine has also been utilized for managing chronic granulomatous foreign body reactions and recurrence of inflammatory filler reactions. Although it can possibly act as an adjuvant treatment option in certain cases that are refractory to other treatment methods, there is not enough information available about the safety and efficacy of colchicine use in PLLA-related nodules [53,54].

In general, through the comparison made, it becomes evident that studies included in this scoping review provide a more refined, PLLA-focused, and detailed understanding of underlying mechanisms than the existing safety literature and PLLA overview papers. The key strength of the present analysis lies in its capacity to connect the onset time, nodules’ characteristics, underlying pathophysiological mechanisms, and therapeutic outcomes into one coherent picture, which cannot be achieved when comparing various products and techniques. The core weakness common to both sets of papers continues to be the limited number of RCTs examining non-dilution therapy.

Limitations and recommendations

However, there were several limitations that could have influenced the interpretation of this scoping review's results. First, a relatively high number of observational studies (case reports, case series, retrospective cohorts, and consensus papers) with a much smaller number of randomized or prospective comparative trials were available in the current literature. Second, high variability across different studies in patient population characteristics, PLLA formulation, type of dilution, technique of injection, time of follow-up, definition of outcomes, and reporting approach made a comparison and further pooling of data nearly impossible.

Another important limitation of this study is associated with the inconsistent incidence, recurrence, and response to therapy rates provided in the literature. Most of the relevant articles had no denominator or lacked standardized diagnostic criteria or a follow-up period to enable estimation of these rates. Another limitation is related to some of the treatment approaches, which were suggested in the literature without solid evidence and were mostly based on expert opinion or general filler granuloma data. While rare vascular and visual complications were considered to be included in the scoping review due to their relevance to recognition and differential diagnosis of complications, they were out of the scope of this study.

With the above considerations notwithstanding, the current review presents an in-depth evidence mapping approach to PLLA-associated nodules by incorporating the onset, clinical presentation, pathophysiology, diagnostics, and treatment into one coherent pathway. Contrasting with filler safety review studies that cover a broad range of fillers, the current study provides a more comprehensive evidence pathway for decision-making in relation to PLLA fillers.

In the future, further studies will need to focus on prospective multi-center studies and randomized controlled trials that assess the efficacy of treatments such as intralesional corticosteroids, 5-fluorouracil, antibiotics, immunomodulatory drugs, and novel adjunctive techniques. It would be advisable to conduct additional studies with the aim of developing diagnostic criteria, grading scales, recurrence predictors, and outcome measures, among others.

## Conclusions

This review synthesizes information from 40 articles published between 2000 and 2026 and proposes an evidence map for the management of nodules associated with PLLA. It shows that nodules related to PLLA represent a heterogeneous complication group comprising various types, with different pathogenesis and management considerations. Prevention through adequate dilution and correct injection technique remains the most evidence-supported approach. This study provides an evidence-informed classification and management pathway consisting of five types and six steps. Observational data form the core of the existing body of knowledge, which mostly includes case reports and case series. Currently, high-quality evidence from RCTs is lacking for intralesional therapies in PLLA nodules. New products, such as porous PLLA microspheres, show lower rates of nodule formation. Moreover, standardizing the reporting of PLLA-related complications in terms of timing, management, and long-term outcomes could enhance the scientific knowledge base.
